# A study of transverse maxillomandibular discrepancy and dental compensation in early mixed dentition with skeletal Class III malocclusion without posterior crossbite

**DOI:** 10.1371/journal.pone.0287343

**Published:** 2023-06-15

**Authors:** Fangming Liu, Haiyun Huang, Xiaoyang Shi, Yi Liu, Dongxu Liu

**Affiliations:** 1 Department of Orthodontics, School and Hospital of Stomatology, Cheeloo College of Medicine, Shandong University & Shandong Key Laboratory of Oral Tissue Regeneration & Shandong Engineering Laboratory for Dental Materials and Oral Tissue Regeneration & Shandong Provincial Clinical Research Center for Oral Diseases, Shandong, China; 2 Department of Implantology, School and Hospital of Stomatology, Cheeloo College of Medicine, Shandong University & Shandong Key Laboratory of Oral Tissue Regeneration & Shandong Engineering Laboratory for Dental Materials and Oral Tissue Regeneration & Shandong Provincial Clinical Research Center for Oral Diseases, Shandong, China; Universidade Federal Fluminense, BRAZIL

## Abstract

**Objective:**

To evaluate transverse maxillomandibular discrepancy and dental compensation in first molar areas in 7- to 9-year-old children with skeletal Class III malocclusion without posterior crossbite using cone-beam computed tomography (CBCT).

**Methods:**

The sample of this retrospective study consisted of 60 children (7 to 9 years old), who were divided into the skeletal Class III malocclusion group (study group, skeletal Class III malocclusion without posterior crossbite, N = 31) and the Class I occlusion group (control group, Class I occlusion with one or two impacted teeth, N = 30). CBCT data were obtained from the database of the Department of Radiology of Hospital of Stomatology, Shandong University. For three-dimensional reconstruction of the head, the dental arch width, basal bone width, and buccolingual inclination angle were measured using MIMICS 21.0 software. Independent-sample *t* tests were used to compare the two groups.

**Results:**

The mean age of the children was 8.18±0.83years. The width of the maxillary basal bone was significantly smaller in the skeletal Class III malocclusion group (59.75 ± 3.14 mm) than in the Class I occlusion group (62.39 ± 3.01 mm) (*P* < 0.01). The mandibular basal bone width was significantly larger in the skeletal Class III malocclusion group (60.00 ± 2.56 mm) than in the Class I occlusion group (58.19 ± 2.42 mm) (*P* < 0.01). The difference in the width of the maxillary and mandibular bases in the skeletal Class III malocclusion group (–0.25 ± 1.73 mm) was significantly different from that in the Class I occlusion group (4.20 ± 1.25 mm) (*P* < 0.01). However, there was no significant difference in the upper or lower dental arch width between the two groups (*P* > 0.05). The buccal inclination of the maxillary molars in the skeletal Class III malocclusion group (31.4° ± 8.9°) was significantly higher than that in the Class I occlusion group (17.64° ± 7.3°) (*P* < 0.01), as was the lingual inclination angle of mandibular molars (45.24° ± 8.3° vs. 37.96° ± 10.18°; *P* < 0.01).

**Conclusion:**

Transverse maxillary and mandibular discrepancies in the posterior area and transverse dental compensation were found in the early mixed dentition of patients with skeletal Class III malocclusion without posterior crossbite. This suggests that even in the absence of posterior crossbite, maxillary expansion can be attempted to correct the maxillomandibular transverse discrepancy.

## Introduction

Skeletal Class III malocclusion is a growth-related clinical craniofacial abnormality that manifests primarily as the lower arch protruding in front of the upper arch [1, 2]. This abnormality establishes itself early in life and is not a self-correcting discrepancy [3–5]. Sagittal dental and skeletal abnormalities can be diagnosed easily via clinical performance or imaging. However, for patients with early mixed dentition without posterior crossbite, transverse maxillomandibular discrepancy can be masked by changes in the inclination of the upper and lower molars. Krishnaswamy [6] noted that although transverse maxillomandibular discrepancies are major components of several forms of malocclusion, crossbite and transverse discrepancies do not form a homologous group; the transverse dimension grows the least and stops growing the soonest by the time the patients are seen. Intervention in the early mixed dentition phase (prepubertal growth phase) is recommended [7–9]. Hence, it is vital to assess the craniofacial skeleton in the transverse dimension as early as possible to identify the need for transverse maxillary expansion and reduce the extent of the burden of severe Class III malocclusion in late adolescence [1, 10].

In clinical practice, the diagnosis of transverse maxillomandibular discrepancies can be difficult and often includes one or more of the following methods: clinical evaluation, dental cast assessment, and radiograph analysis. The advent of cone-beam computed tomography (CBCT) and medical image reconstruction software [11] allows the visualization and analysis of the width of the maxillary and mandibular basal bones and their relationship, the buccolingual inclination of each whole tooth, and their root positions in the alveolar bone [10, 12, 13]. Miner et al. [14] found skeletal discrepancies in the crossbite group by developing a transverse analysis based on CBCT data. Yang et al. [15] found that maxillary first molars exhibited buccal inclination and that adults displayed less inclination than did children in CBCT images. Additionally, Ahn et al. [16] found that transverse dental compensation is closely related to sagittal and transverse skeletal discrepancy in skeletal Class III patients in adults through CBCT. Until now, there have been few studies on transverse maxillomandibular discrepancy and dental compensation with permanent dentition and skeletal Class III malocclusion without posterior crossbite [14, 17], but no research data on transverse maxillomandibular discrepancy and dental compensation in children with early mixed dentition

The objective of this study was to evaluate the transverse maxillomandibular discrepancy and dental compensation in early mixed dentition with skeletal Class III malocclusion without posterior crossbite, in order to provide a theoretical foundation in transverse discrepancy diagnosis and treatment planning.

## Materials and methods

### Subjects

This retrospective study included 7- to 9-year-old children who visited the Hospital of Stomatology, Shandong University from January 2018 to June 2021. Instead of recruiting participants for research, we retrospectively searched CBCT images and identified individual medical records that were archived in the Hospital of Stomatology, Shandong University during the time mentioned above for diagnosis or treatment planning, including planning for orthodontic treatment and surgical removal of impacted supplementary teeth.

All procedures performed in the study were approved by the Ethics Committee of the Hospital of Stomatology, Shandong University (No. 20201209) and were in accordance with the Declaration of Helsinki for research involving human participants. All patients’ parents provided written informed consent.

### Inclusion and exclusion criteria

Sample size estimation was performed using PASS software (Number Cruncher Statistical Systems, version 2000, Kaysville, UT, USA), following the “Two-Sample T-Tests Assuming Equal Variance” calculation method. The used variable was Mx-Mx width. To detect a mean difference of 3.8 mm in the maxillary wide between both groups with a standard deviation of 4.0 mm [18], a test power of 80%, and a significance level of 5%, 19 patients would be needed in each group. To increase test power, even more, all the patients fit the selection criteria were included. At last, the study group (with skeletal Class III malocclusion, N = 31) and the control group (with skeletal Class I occlusion, N = 30) were included, and the final test power reached 95%.

The distribution of sex and age among all samples is shown in Table 1.

**Table 1:** Distribution of sex and age.

Variable	Class III	Class I	Total
Sex, n (%)			
Male	15 (48.4)	17 (56.7)	32 (52.5)
Female	16 (51.6)	13 (43.3)	29 (47.5)
Age (y)			
Average (SD)	8.10 (0.83)	8.27 (0.83)	8.18 (0.83)

Patients in the study group who met the following criteria were included: (1) ANB angle [formed by the subspinale (A), nasion (N), and supramental (B)] less than 0°; anterior teeth couldn’t reach to edge-to-edge relationship when the mandible was retruded; skeletal Class III, symmetry, with Me deviation less than 2 mm;(2) Maxilla to cranial base- Nasion perpendicular to point A (A-Np) less than 0mm [19]; (3) Mandible to cranial base- Pogonion to nasion perpendicular(Pog-Np) more than -6mm [19]; (4) Tweed, who declared that the mandibular incisors should upright in the mandibular anterior alveolus in ideal occlusion, presented the theory that the measurement of mandibular plane angle (FMA) was also essential in demonstrating different growth patterns [20]. Therefore, the FMA—measured between the Frankfort horizontal plane (FH) and the mandibular plane on a lateral cephalogram were used in the criteria. The angle between 22° and 32° (according to Chinese standards) [21] were considered as an average face angle, and the patients meet with this criteria were included.; (5) full eruption of all maxillary and mandibular first permanent molars to the occlusal plane; (6) bilateral first permanent molars with Class III malocclusion; (7) absence of crossbite on permanent molars.

Patients in the control group were selected using the following criteria: (1) 1° < ANB < 4.5° [3]; (2) A-Np about 0mm [19]; (3) Pog-Np between -8mm and -6mm [19]; (4) FMA between 22° and 32°; (5) bilateral first permanent molars with Class I relationship; (6) bilateral maxillary and mandibular first permanent molars fully erupted to the occlusal plane,; (7) no previous orthodontic treatment and approximate symmetry in frontal view and fair profile; (8) anterior crowding, less than 4 mm.

The exclusion criteria were as follows: (1) patients with functional Class III relationship (the mandible could retruded to anterior teeth edge-to-edge position) or a mandibular functional shift; (2) severe dental or maxillofacial deformities, such as cleft lip or palate; (3) any of the first molar has caries, restoration, pulp or periapical disease; (4) abnormal teeth position (one or more permanent teeth missing, premature loss of deciduous teeth).

### Data collection and processing

#### Data collection

All data in this study were obtained using a NewTom 5G CBCT scanner (Verona, Italy). Taking into account radiation doses, safety, and patient protection, the operators followed the As Low As Reasonably Achievable principle before scanning [22, 23] by reducing the standard dose for the echo scan (110 kV, 17.76 mAs), shortening the time of irradiation, and minimizing exposure of the surface of the body. We highlighted the features through which CBCT provided advantages over conventional two-dimensional imaging in paediatric dentistry before orthodontic treatment [24]. Clinical data and records were obtained from the Department of Orthodontics, Hospital of Stomatology, Shandong University.

#### Head position reorientation

**Fig 1:**
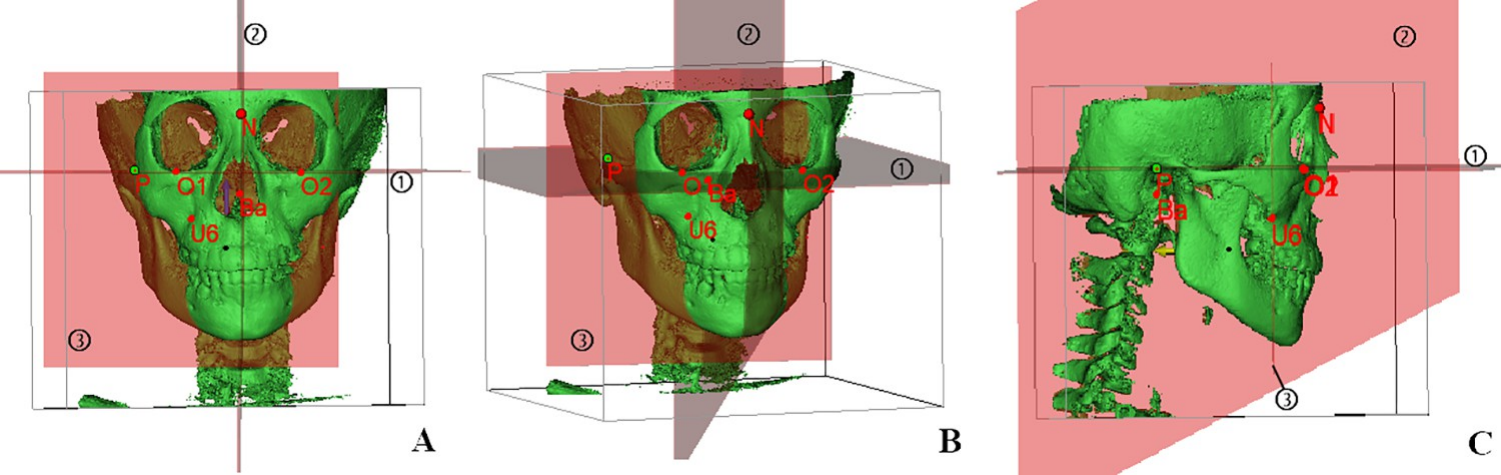
Head position correction. A: Corrected head position on the coronal plane; B: side view of corrected head position. C: Sagittal view of corrected head position.

The CBCT output was obtained in digital imaging and communications in medicine (DICOM) format. Then, the original patient CBCT data were imported into Materialism’s Interactive Medical Image Control System (MIMICS) software (21.0, Leuven, Belgium) to reconstruct a three-dimensional model of the patient’s head, as shown in Fig 1. The landmarks were used as follows:

① Right porion points (P): the most superior point of the right external acoustic meatus.
② Bilateral orbital point (O1 and O2): the most inferior point of the right (O1) and the left (O2) infraorbital rim.
③ Skull base point (Ba): the most anterior point of the great foramen (foramen magnum)
④ Nasal root point (N): the most anterior point of the sutura nasofrontalis.
⑤ Upper molar point (U6): mesiobuccal root tip point of the right upper molar.

The following three reference planes were established using the specified points and planes for head position correction [25]:

① Horizontal reference plane: established through P, O1 and O2.
② Sagittal reference plane: established through Ba and N perpendicular to the horizontal reference plane.
③ Coronal reference plane: established as a plane perpendicular to the horizontal reference plane and the sagittal reference plane, crossing the mesiobuccal root tip point at U6.

The fluoroscopy function on the view menu was used to redefine new coordinates, or the established plane was used as a reference plane to correct the three-dimensional position of the CBCT scan so that the horizontal plane was parallel to the ground plane. Therefore, the head position was reoriented.

Points: P: right porion point; O1: right infraorbital point; O2:left infraorbital point N: nasal root point; Ba: skull base point; U6: mesiobuccal root tip point of right upper molar

Planes:①Horizontal reference plane(FH): established through P, O1 and O2.②Sagittal reference plane(MSP): established through Ba and N perpendicular to the horizontal reference plane.③Coronal reference plane(VRP): established as a plane perpendicular to the horizontal reference plane and the sagittal reference plane, crossing the mesiobuccal root tip point at U6.

### Measurements

#### Width

**Fig 2:**
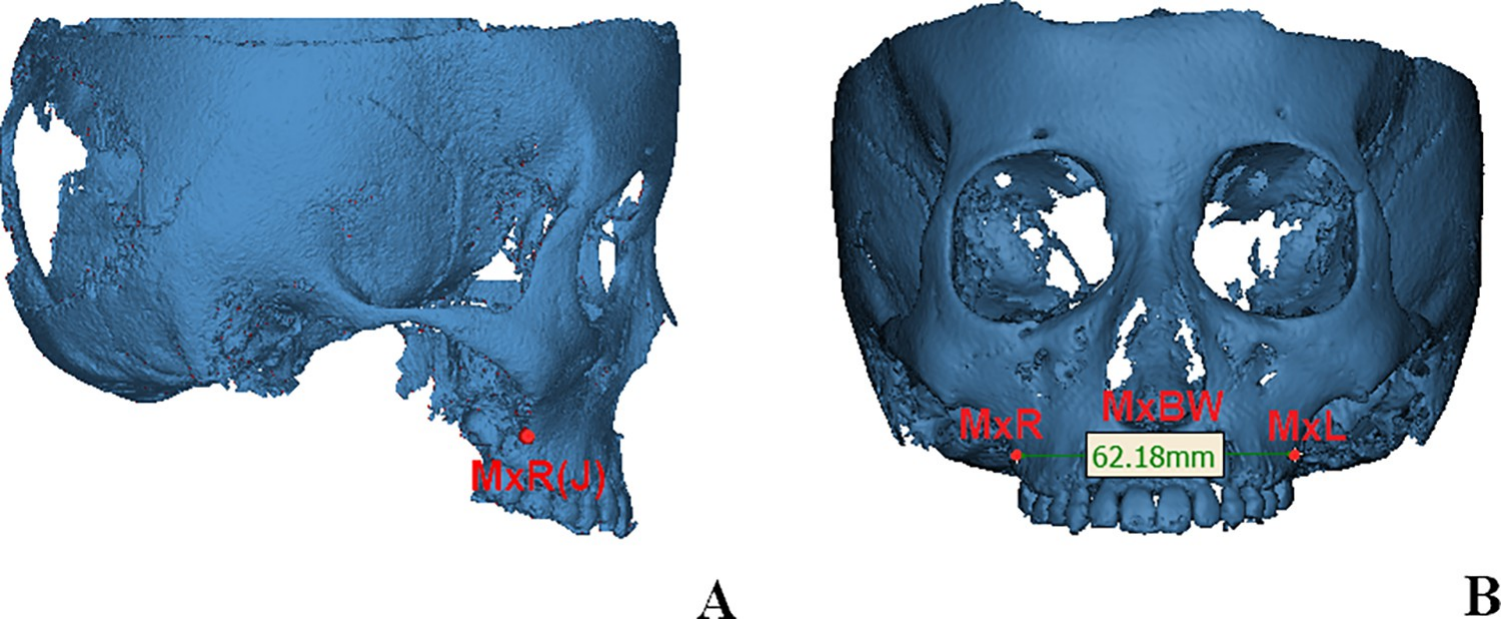
Locations of points and measurements in maxillary basal bone width. A. On the coronal view, the location of the maxillary measurement points (MxR and MxL) were on the right and left intersection points of the maxillary tuberosity outline and the zygomatic buttress; on the sagittal view, they were in accordance with the position of maxillary first molar. B The distance between MxR and MxL corresponded to the width of the maxillary basal bone width (MxBW).

**Fig 3:**
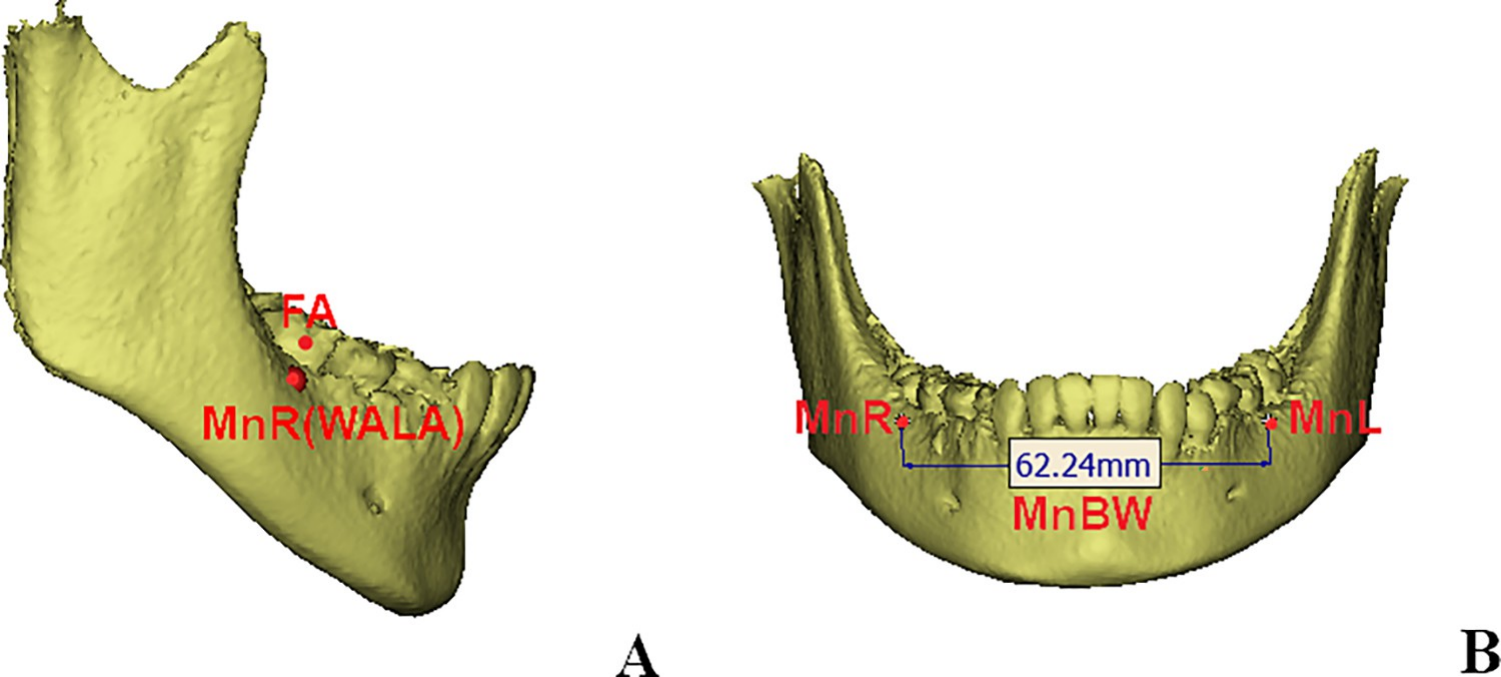
Locations of points and measurements in mandibular basal bone width. A. On the axial view, the most prominent points of the buccal alveolar process were allocated, the vertical positions were in accordance with the center of resistance points of the mandibular first molars. They were defined as the right and left mandibular width measurement points (MnR and MnL). B. The distance between MxR and MxL was in correspondence with the widths of the mandibular basal bone widths (MnBW).

**Fig 4:**
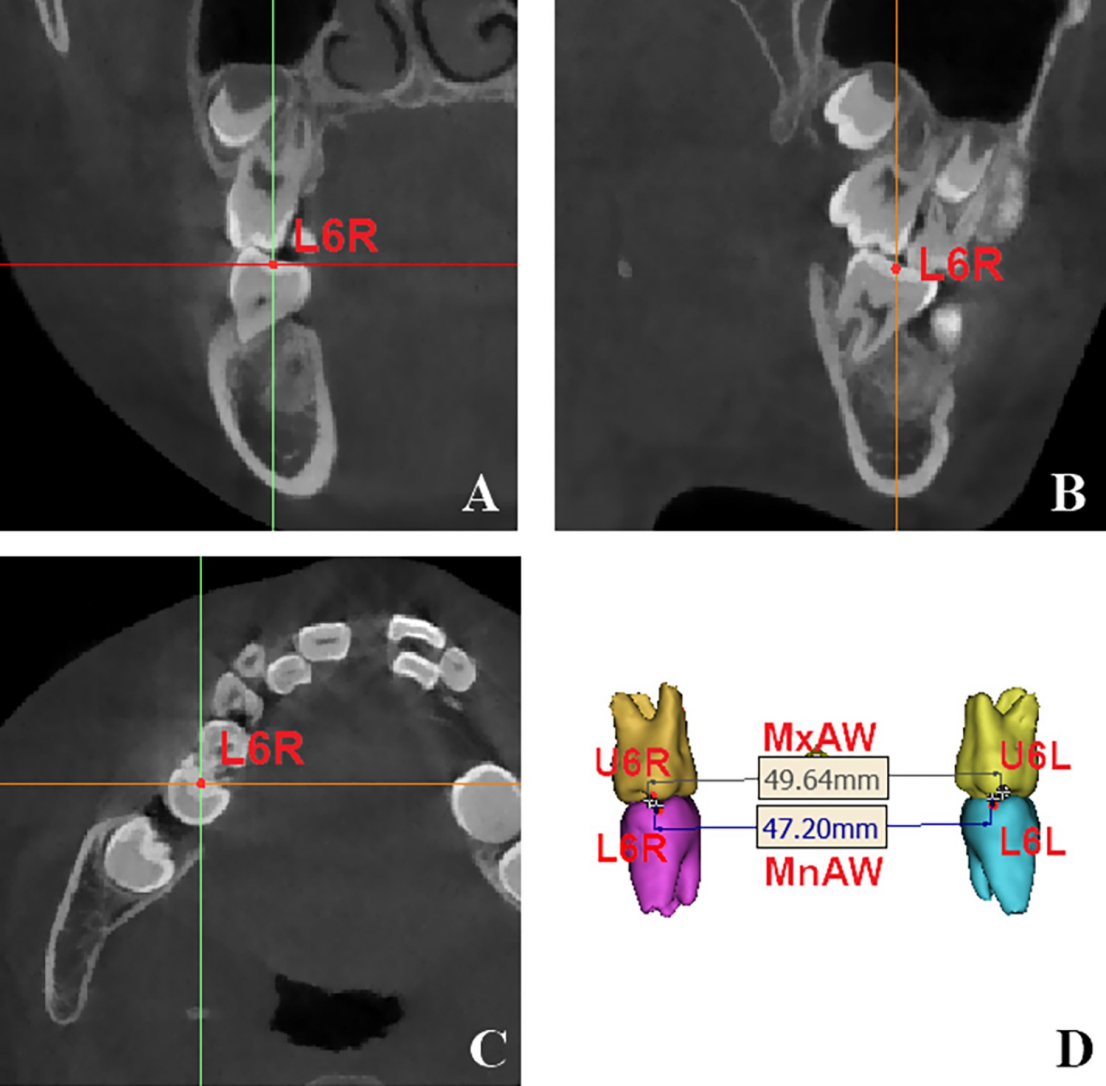
Location of marked points in the maxillary and mandibular arches. A~C: Central fossa of the right maxillary first molar on the coronal, sagittal and axial planes; D: Maxillary arch width (MxAW): distance between the two central fossa of the maxillary first molars (U6R-U6L); Mandibular arch width (MnAW): distance between the two central fossa of the mandibular first molars (L6R-L6L).

We established another analysis system using the “Measure and Analyze—Analysis Overview” menu; the system was built to specify eight positioning points, and the type of measurement was set as distance (2 points). The eight points and four distances were defined as follows:

(1) On the coronal view, the location of the maxillary measurement points (MxR and MxL) were on the right and left intersection points of the maxillary tuberosity outline and the zygomatic buttress; on the sagittal view, they were in accordance with the position of maxillary first molar. The distance between them corresponded to the width of the maxillary basal bone width (MxBW) [24] (Fig 2).
(2) On the axial view, the most prominent points of the buccal alveolar process were allocated, the vertical positions were in accordance with the center of resistance points of the mandibular first molars. They were defined as the right and left mandibular width measurement points (MnR and MnL). The distance between them was in correspondance with the widths of the mandibular basal bone widths (MnBW). (Fig 3).
(3) Points U6R and U6L were the central fossa of the right and left maxillary first molars, and the dental arch widths (MxAW) was the distance between them; the central fossa of the right and left mandibular first molars were allocated at Points L6R and L6L, the distance between them was defined as mandibular dental arch width (MnAW) [26] (Fig 4).

After the positioning was completed, the “Measure and Analyze” function of MIMICS software automatically obtained the measurements of each width in three-dimensional space. The difference between the basal bone width was defined as the MxBW minus MnBW, and the difference between the dental arch width was MxAW minus MnAW. The value was considered positive when width of the maxilla is larger than that of the mandible, and negative when the former is smaller than the latter.

#### Angle

**Fig 5:**
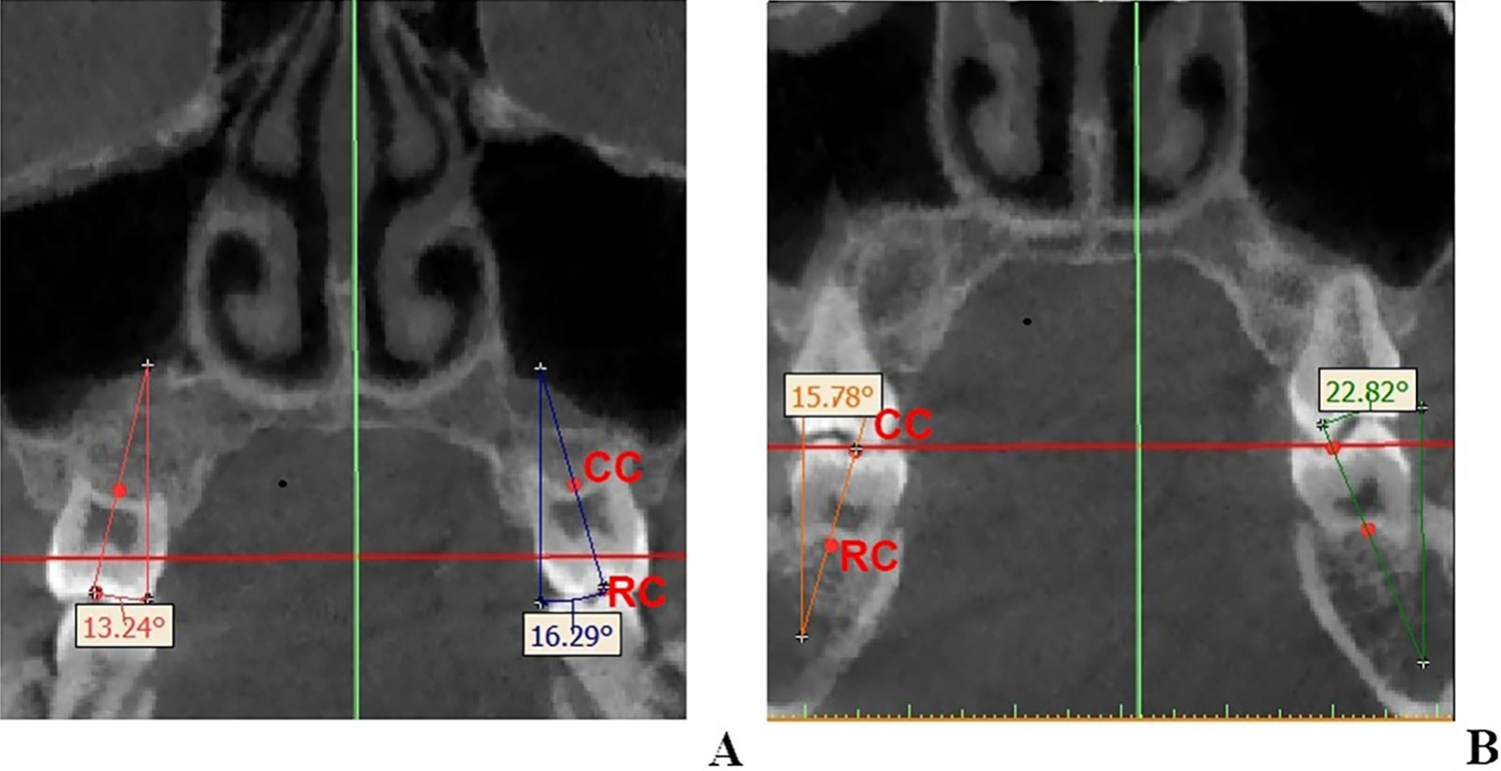
Inclination measurements for the maxillary and mandibular first molars. A: Angles of maxillary first molars on coronal view. B: Angles of mandibular first molars on coronal view.

The central point of the clinical crown (CC) and the root centre (RC) point were defined by the method proposed by Alkhatib [27]. At first, the root centre (RC) point was defined as the center of root furcation, which were located and calibrated in three dimensions. Then the coronal cut where the RC located was chosen; the midpoint of the buccal-lingual width of the crown was located and defiend as the central point of the clinical crown (CC). The long axis of the tooth (LAT) was defined as the line connecting CC and RC points. The coronal plane passing through the CC point and the RC point was selected as the measuring plane, and the angle between the LAT and the vertical line perpendicular to the FH was measured. The maxillary LAT was considered positive on the buccal side of the vertical line and negative on the lingual side; while the mandibular LAT was considered positive on the lingual side of the vertical line and negative on the buccal side (Fig 5).

The central point of the clinical crown (CC) and the root centre (RC) point were defined by the method proposed by Alkhatib [27]. At first, the root centre (RC) point was defined as the center of root furcation, which were located and calibrated in three dimensions. Then the coronal cut where the RC located was chosen; the midpoint of and buccal-lingual width of the crown was located and defiend as the central point of the clinical crown (CC). The long axis of the tooth (LAT) was defined as the line connecting CC and RC points. The coronal plane passing through the CC point and the RC point was selected as the measuring plane, and the angle between the LAT and the vertical line perpendicular to the FH was measured. The maxillary LAT was considered positive on the buccal side of the vertical line and negative on the lingual side; while the mandibular LAT was considered positive on the lingual side of the vertical line and negative on the buccal side.

#### Repeated data measurement

Using MIMICS software, the experimenter used the same method to repeat the measurements again after a two-week interval. The intragroup correlation coefficient (ICC) of the data obtained from the two measurements was calculated to evaluate the reliability The random errors (RE) were evaluated using Dahlberg formula [28]: $D=\sqrt{\sum (d^{2}/2n)}$ (where *d* indicates deviations between the 2 measurements, and *n* indicates number of paired objects). The final values of the aforementioned measurements were averaged.

### Statistical analysis

Standard descriptive statistics, including means and standard deviations, were calculated for each measurement. The normality of the outcome data of widths and angles was examined using the Kolmogorov–Smirnov test with SPSS 25.0 (IBM SPSS Statistics 25.0) software. As all data conformed to a normal distribution, independent-sample *t* tests were performed to compare the two groups. A *P* value <0.05 indicated statistical significance.

## Results

By analysing, the intraclass correlation coefficients of repeated measurements were all greater than 0.85, indicating good reliability. The random error of each of the measurements were shown as follows: the error of angle measurements ranged from 0.47° to 0.57°, whereas the error of the width measurements ranged from 0.38 mm to 0.46 mm.

A comparison of the maxillary base width (MxBW) and mandibular base width (MnBW) between the two groups showed a significantly narrower maxillary molar area and a significantly wider mandibular molar area in skeletal Class III malocclusion without posterior crossbite than in Class I occlusion (*P* < 0.01). The difference in the width of the maxillary and mandibular bases was 4.20 ±1.25 mm in Class I occlusion and –0.25 ± 1.73 mm in skeletal Class III malocclusion without posterior crossbite, and the difference was significant (*P* < 0.01) (Table 2).

**Table 2:** Comparison of the widths of the maxillary and mandibular bases in the two groups.

Groups	Class Ⅲ	Class Ⅰ	*t*	*P*
MxBW	59.75 ± 3.14	62.39 ± 3.01	3.356	0.001**
MnBW	60.00 ± 2.56	58.19 ± 2.42	–2.833	0.006**
MxBW–MnBW	–0.25 ± 1.73	4.20 ± 1.25	11.488	0.000**

*P < 0.05; **P < 0.01.

MxBW, maxillary base width; MnBW, mandibular base width

There was no significant difference in the maxillary dental arch width (MxAW), the mandibular dental arch width (MnAW), or the difference between the two (MxAW–MnAW) between the two groups (*P* > 0.05) (Table 3).

**Table 3:** Comparison of the widths of the maxillary and mandibular arches in the two groups.

Groups	Class Ⅲ	Class Ⅰ	*t*	*P*
MxAW	47.74 ± 2.81	48.20 ± 3.53	0.560	0.578
MnAW	47.49 ± 3.00	48.42 ± 2.25	1.373	0.175
MxAW–MnAW	0.26 ± 1.75	–0.22 ± 2.32	–0.911	0.366

*P < 0.05; **P < 0.01.

MxAW, maxillary dental arch width; MnAW, mandibular base width.

The analysis also revealed no significant difference in the indexes between the sexes in the two groups (P > 0.05) (Table 4).

**Table 4:** Comparison of the widths and inclination angles by sex.

**Class I**	*P*	0.982	0.388	0.422	0.182	0.266	0.297	0.279	0.436
	*t*	0.023	0.878	-0.815	1.369	1.134	1.062	1.104	0.790
	Female	48.18±2.66	48.01±2.42	0.17±2.36	61.55±3.02	57.62±2.41	3.93±1.43	15.97±6.84	36.27±11.24
	Male	48.21±4.15	48.74±2.13	-0.53±232	63.04±2.92	58.63±2.40	4.42±1.10	18.93±7.58	39.25±9.42
**Class III**	*P*	0.682	0.835	0.763	0.418	0.882	0.208	0.605	0.462
	*t*	0.414	0.210	0.304	-0.821	-0.149	-1.288	0.523	0.745
	Female	47.54±3.04	47.38±3.33	0.16±1.97	60.20±3.82	60.06±2.96	0.13±1.74	30.58±7.51	44.16±7.90
	Male	47.96±2.63	47.61±2.71	0.36±1.56	59.27±2.25	59.93±2.16	-0.66±1.68	32.28±10.38	46.40±8.83
**Group**		MxAW	MnAW	MxAW–MnAW	MxBW	MnBW	MxBW–MnBW	UMIA	LMIA

The upper molar inclination angle (UMIA) and lower molar inclination angle (LMIA) of the buccolingual orientation of the left and right molars were not significantly different (*P* > 0.05). Therefore, data from the left and right sides were combined for subsequent comparisons between the groups.

The UMIA in the skeletal Class III malocclusion and Class I occlusion groups was 31.4 ± 8.9° and 17.64 ± 7.3°, respectively; the difference between the two groups was significant (*P* < 0.01). The LMIA was higher in skeletal Class III malocclusion without posterior crossbite (45.24 ± 8.3°) than in Class I occlusion (37.96 ± 10.18°, *P*< 0.01) (Table 5). There were no significant differences in the two aforementioned indexes between the sexes in the two groups (*P* > 0.05) (Table 4).

**Table 5:** Comparison of inclination angles in the two groups.

Group	Class Ⅲ	Class Ⅰ	*t*	*P*
UMIA	31.40 ± 8.90	17.64 ± 7.30	–6.589	0.000**
LMIA	45.24 ± 8.30	37.96 ± 10.18	–3.068	0.003**

*P < 0.05; **P < 0.01.

LMIA, lower molar inclination angle; UMIA, upper molar inclination angle.

## Discussion

The results of our study show that in the skeletal Class III malocclusion group, the maxillary basal bone width was significantly narrower (*P* < 0.01) and the mandibular basal bone width was significantly wider (*P* < 0.01) than that in the Class I occlusion group, which is similar to results reported for a population with permanent dentition by our previous studies [25, 29]. This indicates that patients with skeletal Class III malocclusion without posterior crossbite in the early stage of mixed dentition also suffer from insufficient maxillary width and/or excessive mandibular width; in other words, uncoordinated maxillary and mandibular widths emerge during this period. However, no significant difference in the upper or lower arch width between the two groups were found in our study. The compensation of the maxillary and maxillary permanent molars before orthodontic treatment is present in the period of mixed dentition in patients with skeletal Class III malocclusion. Therefore, during clinical diagnosis, the dental arch widths cannot fully reflect the coordination of the transverse widths of the upper and lower jaws; indeed, measurement of the width of the maxillary and mandibular basal bone might be more clinically significant.

Wilson [30] was the first to report the lateral inclination of the grinding teeth, with the lower teeth inclined lingually and the upper teeth inclined buccally. It is important to determine the appropriate amount of buccolingual tooth inclination for adequate function and to quantify it so that treatment goals are well supported by evidence. Yang et al. [15] reported that the normal buccal inclination of the maxillary and mandibular first molars in children with untreated Class I occlusion was 21.1° ± 9.5° and 34.9° ± 11°, respectively, similar to that in the Class I occlusion patients in our study. Alkhatib and Chung [27] showed that the maxillary buccal and mandibular lingual inclination in normal adults was 4.9° and 12.6°, respectively. Marshall et al. [31] found that the buccolingual inclination of the molars gradually decreased with age, explaining the difference between their research and our measurement results.

The result of our study showed that the first permanent maxillary molars of children with skeletal Class III malocclusion without posterior crossbite compensated more for the buccal inclination than those with Class I occlusion (*P* < 0.01), and the mandibular first molars were more lingual (*P* < 0.01). McNamara [17] reported that the buccal inclination of the maxillary molars and the depth of the Wilson curve of the mandibular molars tended to increase when the posterior teeth had no crossbite but the width of the maxilla was insufficient. Miner et al. [14] used CBCT in the analysis of the transverse dimension in mixed or permanent dentition. They found that within the clinical non-crossbite group, a significant number of patients were revealed to have skeletal transverse jaw discrepancy that had been masked by dental compensation; these results are in accordance with the results of our study.

In this study, the maxillary and mandibular basal bone widths were measured, and the measurement points were located with reference to the first molars. Based on former studies, it is common to use teeth position to locate bone reference, but there is no doubt that teeth position could affect the result of basal bone width measurement. When the molars moving mesially, the corresponding basal bone width would be narrower. On the other hand, when the molars moving distally, the basal bone width would be wider. One or more permanent teeth missing, premature loss of deciduous teeth would affect the position of the first molars. In this study, patients with abnormal teeth position were excluded, thus the influence would be minimized.

The information obtained from CBCT imaging provides several substantial advantages. For example, CBCT imaging provides accurate measurements, improves localization of impacted teeth, provides visualization of airway abnormalities comparison to conventional radiograph. Moreover, CBCT imaging involves only a minimal increase in radiation dose relative to combined diagnostic modern digital panoramic and cephalometric imaging. But CBCT is not a standard method of diagnosis in Orthodontics. most frequent indication of CBCT in Orthodontics, includes retained/impacted permanent teeth; severe craniofacial anomalies; and so on. In this study, the indications in study group (skeletal Class III malocclusion) were severe craniofacial anomalies; in control group were at least one impacted tooth and the As Low As Reasonably Achievable principle [22, 23] was conducted in our study.

There are some limitations of this study. First of all, considering the large variations in growth and different types of dentition replacement among 7- and 9-year-old children, the sample size of the study is limited. Furthermore, the population is limited to Chinese, therefore the results couldn’t be generalized to other populations. Larger sample size and different populations are needed in further study. Second, longitudinal study is preferred to give more clinical evidence. But leaving a skeletal Class III patients untreated is against ethics. Data acquired from previous studies could be used, and future study should be carefully designed. In addition, the classification of skeletal Class III malocclusion is complex. This study was not specified whether class III was due to mandibular prognathism, maxillary retrognathism or a combination of both. Skeletal Class Ⅲ patients with different pathogenesis should be explored for better results.

## Conclusions

The main findings were as follows:

(1) Abnormal transverse development of the maxillary and mandibular basal bones in patients with skeletal Class III malocclusion without posterior crossbite is present in the early stage of mixed dentition. RME might be the most efficient and effective treatment for transverse problems in Class III patients in early mixed dentition stage.
(2) In patients with mixed dentition, the inclination of the upper and lower molars is higher among those with skeletal Class III malocclusion without posterior crossbite than among those with Class I occlusion due to compensation for maintaining occlusal contact.

## Supporting information

S1 Checklist STROBE statement—checklist of items that should be included in reports of observational studies. (DOCX)
