# Supplementary material for: A study of transverse maxillomandibular discrepancy and dental compensation in early mixed dentition with skeletal Class III malocclusion without posterior crossbite
Source: PLoS One. 2023 Jun 15;18(6):e0287343. doi: 10.1371/journal.pone.0287343 (PMC10270617; doi:10.1371/journal.pone.0287343)
Supplement: S1 Checklist — (DOCX) [file pone.0287343.s001.docx]

STROBE Statement—checklist of items that should be included in reports of observational studies

|  | Item No. | Recommendation | Page  No. | Relevant text from manuscript |
| --- | --- | --- | --- | --- |
| **Title and abstract** | 1 | (*a*) Indicate the study’s design with a commonly used term in the title or the abstract | 2 | The sample of this retrospective study |
|  |  | (*b*) Provide in the abstract an informative and balanced summary of what was done and what was found | 2 | **Methods:** The sample of this retrospective study consisted of 60 children (7 to 9 years old), who were divided into the skeletal Class III malocclusion group (study group, skeletal Class III malocclusion without posterior crossbite, N = 30) and the Class I occlusion group (control group, Class I occlusion with one or two impacted teeth, N = 30). CBCT data were obtained from the database of the Department of Radiology of Hospital of Stomatology, Shandong University. For three-dimensional reconstruction of the head, the dental arch width, basal bone width, and buccolingual inclination angle were measured using MIMICS 21.0 software. Independent-sample *t* tests were used to compare the two groups.  **Results:** The mean age of the children was 8.15±0.82 years. The width of the maxillary basal bone was significantly smaller in the skeletal Class III malocclusion group (59.75 ± 3.14 mm) than in the Class I occlusion group (62.39 ± 3.01 mm) (*P* < 0.05). The mandibular basal bone width was significantly larger in the skeletal Class III malocclusion group (60.00 ± 2.56 mm) than in the Class I occlusion group (58.19 ± 2.42 mm) (*P* < 0.05). The difference in the width of the maxillary and mandibular bases in the skeletal Class III malocclusion group (–0.25 ± 1.73 mm) was significantly different from that in the Class I occlusion group (4.20 ± 1.25 mm) (*P* < 0.05). However, there was no significant difference in the upper or lower dental arch width between the two groups (*P* > 0.05). The buccal inclination of the maxillary molars in the skeletal Class III malocclusion group (31.4° ± 8.9°) was significantly higher than that in the Class I occlusion group (17.64° ± 7.3°) (*P* < 0.01), as was the lingual inclination angle of mandibular molars (45.24° ± 8.3° vs. 37.96° ± 10.18°; *P* < 0.05). |
| Introduction | | | |  |
| Background/rationale | 2 | Explain the scientific background and rationale for the investigation being reported | 3-4 | Skeletal Class III malocclusion is a growth-related clinical craniofacial abnormality that manifests primarily as the lower arch protruding in front of the upper arch[1]. This abnormality establishes itself early in life and is not a self-correcting discrepancy[2,3]. Sagittal dental and skeletal abnormalities can be diagnosed easily via clinical performance or imaging. However, for patients with early mixed dentition without posterior crossbite, transverse maxillomandibular discrepancy can be masked by changes in the inclination of the upper and lower molars. Krishnaswamy[4] noted that although transverse maxillomandibular discrepancies are major components of several forms of malocclusion, crossbite and transverse discrepancies do not form a homologous group; the transverse dimension grows the least and stops growing the soonest by the time the patients are seen. Intervention in the early mixed dentition phase (prepubertal growth phase) is recommended[5,6]. The prepubertal treatment of Class III malocclusion with transverse maxillomandibular discrepancy with rapid maxillary expansion (RME) yields favourable growth correction in both the maxilla and mandible[6]. In particular, RME can promote adaptive growth of the maxilla, synchronous development of the upper and lower jaws, and more stable treatment effects, reducing the need for later complex correction. Hence, it is vital to assess the craniofacial skeleton in the transverse dimension as early as possible to identify the need for transverse maxillary expansion and reduce the extent of the burden of severe Class III malocclusion in late adolescence[7,8].  In the clinic, the diagnosis of transverse maxillomandibular discrepancies can be difficult and often includes one or more of the following methods: clinical evaluation, dental cast assessment, and posteroanterior (PA) cephalometric analysis. In the 1990s, the PA cephalogram was considered the most readily available and reliable radiograph for evaluating transverse skeletal dysplasia. However, the accuracy of the measurement results is affected by errors in and magnification of the patient’s head position and measurement points[9].  With the advent of cone-beam computed tomography (CBCT) and medical image reconstruction software, images became better correlated with bone skulls, and radiographic diagnostic methods became more valuable[10]. These tools allow the visualization and analysis of the width of the maxillary and mandibular basal bones and their relationship, the buccolingual inclination of each whole tooth, and their root positions in the alveolar bone[8,11,12]. Miner et al[13] found skeletal discrepancies in the crossbite group by developing a transverse analysis based on CBCT data. Yang et al[14] found that maxillary first molars exhibited buccal inclination and that adults displayed less inclination than did children in CBCT images. Additionally, Ahn et al[15] found that transverse dental compensation is closely related to sagittal and transverse skeletal discrepancy in skeletal Class III patients in adults through CBCT. Until now, there have been few studies on transverse maxillomandibular discrepancy and dental compensation in children with early mixed dentition and skeletal Class III malocclusion without posterior crossbite. |
| Objectives | 3 | State specific objectives, including any prespecified hypotheses | 4 | Our study examined 7- to 9-year-old children with skeletal Class III malocclusion without posterior crossbite, and children in the same age range with Class I occlusion with impacted teeth were selected as controls for comparisons. The study outcomes were the basal bone width, dental arch width and buccolingual inclination angle as determined by CBCT reconstruction. The results will provide a theoretical foundation for RME in children with early mixed dentition and skeletal Class III malocclusion even in the absence of posterior crossbite. |
| Methods | | | |  |
| Study design | 4 | Present key elements of study design early in the paper | 5 | The samples were divided into the study group (with skeletal Class III malocclusion, N=30) and the control group (with skeletal Class I occlusion, N=30). The distribution of sex and age among all samples is shown in Table 1. |
| Setting | 5 | Describe the setting, locations, and relevant dates, including periods of recruitment, exposure, follow-up, and data collection | 4-5 | This retrospective study included 7- to 9-year-old children who visited the Hospital of Stomatology, Shandong University from January 2018 to June 2021. Instead of recruiting participants for research, we retrospectively searched CBCT images and identified individual medical records that were archived in the Hospital of Stomatology, Shandong University during the time mentioned above for diagnosis or treatment planning, including planning for orthodontic treatment and surgical removal of impacted supplementary teeth. |
| Participants | 6 | (*a*) *Cohort study*—Give the eligibility criteria, and the sources and methods of selection of participants. Describe methods of follow-up  *Case-control study*—Give the eligibility criteria, and the sources and methods of case ascertainment and control selection. Give the rationale for the choice of cases and controls  *Cross-sectional study*—Give the eligibility criteria, and the sources and methods of selection of participants | 4-5;5-6 | Case-control study—we retrospectively searched CBCT images and identified individual medical records that were archived in the Hospital of Stomatology, Shandong University during the time mentioned above for diagnosis or treatment planning, including planning for orthodontic treatment and surgical removal of impacted supplementary teeth.  The samples were divided into the study group (with skeletal Class III malocclusion, N=30) and the control group (with skeletal Class I occlusion, N=30). The distribution of sex and age among all samples is shown in Table 1.  Patients in the study group who met the following criteria were included: (1) ANB angle [formed by the subspinale (A), nasion (N), and supramental (B)] less than 0°; anterior crossbite and a lack of an edge-to-edge bite on the anterior teeth when the mandible was in the retruded contact position; (2) an average face angle [a mandibular plane angle (FMA)—measured between the Frankfort horizontal plane (FH) and the mandibular plane on a lateral cephalogram by Downs analysis—between and including 22° and 32° according to Chinese standards][17]; (3) full eruption of all maxillary and mandibular first permanent molars to the occlusal plane, without pulp disease or periapical periodontitis; (4) bilateral first permanent molars with mesio-occlusion; (5) absence of crossbite on permanent molars; and (6) no severe dental or maxillofacial deformities, such as cleft lip or palate.  Patients in the control group were selected using the following criteria: (1) 1° < ANB < 4.5°[18]; (2) FMA between 22° and 30°; (3) bilateral first permanent molars with neutrocclusion; (4) bilateral maxillary and mandibular first permanent molars fully erupted to the occlusal plane, without pulp disease or periapical periodontitis; (5) no previous orthodontic treatment and approximate consistency between the front and side appearances; (6) slightly crowded dentition or no more than two impacted teeth except the molars; and (7) no history of major oral or maxillofacial disease. |
|  |  | (*b*) *Cohort study*—For matched studies, give matching criteria and number of exposed and unexposed  *Case-control study*—For matched studies, give matching criteria and the number of controls per case |  |  |
| Variables | 7 | Clearly define all outcomes, exposures, predictors, potential confounders, and effect modifiers. Give diagnostic criteria, if applicable | 5-6 | Patients in the study group who met the following criteria were included: (1) ANB angle [formed by the subspinale (A), nasion (N), and supramental (B)] less than 0°; anterior crossbite and a lack of an edge-to-edge bite on the anterior teeth when the mandible was in the retruded contact position; (2) an average face angle [a mandibular plane angle (FMA)—measured between the Frankfort horizontal plane (FH) and the mandibular plane on a lateral cephalogram by Downs analysis—between and including 22° and 32° according to Chinese standards][17]; (3) full eruption of all maxillary and mandibular first permanent molars to the occlusal plane, without pulp disease or periapical periodontitis; (4) bilateral first permanent molars with mesio-occlusion; (5) absence of crossbite on permanent molars; and (6) no severe dental or maxillofacial deformities, such as cleft lip or palate.  Patients in the control group were selected using the following criteria: (1) 1° < ANB < 4.5°[18]; (2) FMA between 22° and 30°; (3) bilateral first permanent molars with neutrocclusion; (4) bilateral maxillary and mandibular first permanent molars fully erupted to the occlusal plane, without pulp disease or periapical periodontitis; (5) no previous orthodontic treatment and approximate consistency between the front and side appearances; (6) slightly crowded dentition or no more than two impacted teeth except the molars; and (7) no history of major oral or maxillofacial disease. |
| Data sources/ measurement | 8* | For each variable of interest, give sources of data and details of methods of assessment (measurement). Describe comparability of assessment methods if there is more than one group | *6-9* | *The CBCT output was obtained in digital imaging and communications in medicine (DICOM) format. Then, the original patient CBCT data were imported into Materialism’s Interactive Medical Image Control System (MIMICS) software (21.0, Leuven, Belgium) to reconstruct a three-dimensional model of the patient’s head, as shown in Figure 1. The landmarks were used as follows*  *① Bilateral porion points (PL and PR): the most superior point of each external acoustic meatus.*  *② Orbital point (O): the most inferior point of the infraorbital rim.*  *③ Skull base point (Ba): the most anterior point of the great foramen (foramen magnum)*  *④ Nasal root point (N): the most anterior point of the sutura nasofrontalis.*  *⑤ Upper molar point (U6): mesiobuccal root tip point of the right upper molar.*  *The following three reference planes were established using the specified points and planes for head position correction[22]:*  *① Horizontal reference plane: established through PL, PR and O.*  *② Sagittal reference plane: established through Ba and N perpendicular to the horizontal reference plane.*  *③ Coronal reference plane: established as a plane perpendicular to the horizontal reference plane and the sagittal reference plane, crossing the mesiobuccal root tip point at U6.*  *The fluoroscopy function on the view menu was used to redefine new coordinates, or the established plane was used as a reference plane to correct the three-dimensional position of the CBCT scan so that the horizontal plane was parallel to the ground plane.*  *We established another analysis system using the “Measure and Analyze - Analysis Overview” menu; the system was built to specify eight positioning points, and the type of measurement was set as distance (2 points). The eight points and four distances were defined as follows:*  *(1) The corresponding bone is at the level of the mesiobuccal root tip of the left and right maxillary first molars in the three-dimensional view at Points 1 and 2, and the distance between the two points corresponds to the width of the maxillary basal arch[21] (Fig. 2).*  *(2) The distance between the osseous cortical bone corresponding to the WALA ridge at the left and right mandibular first molars is measured in the three-dimensional view between Points 3 and 4; this distance corresponds to the width of the mandibular basal arch (Fig. 3).*  *(3) The central fossa of the left and right maxillary first molars is located in the three-dimensional view at Points 5 and 6, corresponding to the width of the maxillary arch; the central fossa of the left and right mandibular first molars, at Points 7 and 8, correspond to the width of the mandibular arch[23] (Fig. 4).*  *After the positioning was completed, the “Measure and Analyze” function of MIMICS software automatically obtained the measurements of each width in three-dimensional space (Fig. 5). The difference between the base width was defined as the mandible base was subtracted from the maxilla base, and the difference between the width of the maxillary arch and the mandibular arch was obtained. The value was considered positive when width of the maxilla is larger than that of the mandible and negative when the former is smaller than the latter.*  *The FH was kept parallel to the ground, and the central point of the clinical crown (CC) was defined by the method proposed by Alkhatib[24] such that the sagittal plane divided the crown equally in the proximal and distal directions and the coronal plane in the labial (buccal) and lingual directions. The 1-mm space under the root bifurcation was defined as the root centre (RC) point. The long axis of the tooth (LAT) was defined as the line connecting the tooth CC and RC points. The coronal plane passing through the CC point and the RC point perpendicular to the FH was selected as the measuring plane, and the angle between the LAT and the vertical line perpendicular to the FH was measured. The maxillary LAT was considered positive on the buccal side of the vertical line and negative on the lingual side of the vertical line, while the mandibular LAT was considered positive on the lingual side of the vertical line and negative on the buccal side (Fig. 6).* |
| Bias | 9 | Describe any efforts to address potential sources of bias | 9 | Using MIMICS software, the experimenter used the same method to repeat the measurements again after a two-week interval. The intragroup correlation coefficient (ICC) of the data obtained from the two measurements was calculated assess systematic and random errors, and reliability analysis was performed. The final values of the aforementioned measurements were averaged. The intraclass correlation coefficients of repeated measurements were all greater than 0.85. The results showed that the experimental method had good repeatability and high reliability. |
| Study size | 10 | Explain how the study size was arrived at | 5 | The analysis revealed that a sample size of n = 30 patients per group was needed to achieve a power of 80% at an α= 0.05 significance level to detect statistically significant differences between the groups, Sample size estimation was performed using PASS software (Number Cruncher Statistical Systems, version 2000, Kaysville, UT, USA). |

Continued on next page

| Quantitative variables | 11 | Explain how quantitative variables were handled in the analyses. If applicable, describe which groupings were chosen and why | 9 | Standard descriptive statistics, including means and standard deviations, were calculated for each measurement. The normality of the outcome data of widths and angles was examined using the Kolmogorov–Smirnov test with SPSS 25.0 (IBM SPSS Statistics 25.0) software. As all data conformed to a normal distribution, independent-sample t tests were performed to compare the two groups. A P value <0.05 indicated statistical significance. |
| --- | --- | --- | --- | --- |
| Statistical methods | 12 | (*a*) Describe all statistical methods, including those used to control for confounding | 9 | Standard descriptive statistics, including means and standard deviations, were calculated for each measurement. The normality of the outcome data of widths and angles was examined using the Kolmogorov–Smirnov test with SPSS 25.0 (IBM SPSS Statistics 25.0) software. As all data conformed to a normal distribution, independent-sample t tests were performed to compare the two groups. A P value <0.05 indicated statistical significance. |
|  |  | (*b*) Describe any methods used to examine subgroups and interactions |  |  |
|  |  | (*c*) Explain how missing data were addressed |  | No data loss issues |
|  |  | (*d*) *Cohort study*—If applicable, explain how loss to follow-up was addressed  *Case-control study*—If applicable, explain how matching of cases and controls was addressed  *Cross-sectional study*—If applicable, describe analytical methods taking account of sampling strategy | 5 | Case-control study—The samples were divided into the study group (with skeletal Class III malocclusion, N=30) and the control group (with skeletal Class I occlusion, N=30). The distribution of sex and age among all samples is shown in Table 1. |
|  |  | (*e*) Describe any sensitivity analyses |  | No exist any sensitivity analyses |
| Results | | | | |
| Participants | 13* | (a) Report numbers of individuals at each stage of study—eg numbers potentially eligible, examined for eligibility, confirmed eligible, included in the study, completing follow-up, and analysed | 5 | with skeletal Class III malocclusion, N=30  with skeletal Class I occlusion, N=30 |
|  |  | (b) Give reasons for non-participation at each stage |  | Excluded: No satisfied the inclusion criteria consent  Excluded: No informed consent |
|  |  | (c) Consider use of a flow diagram |  | 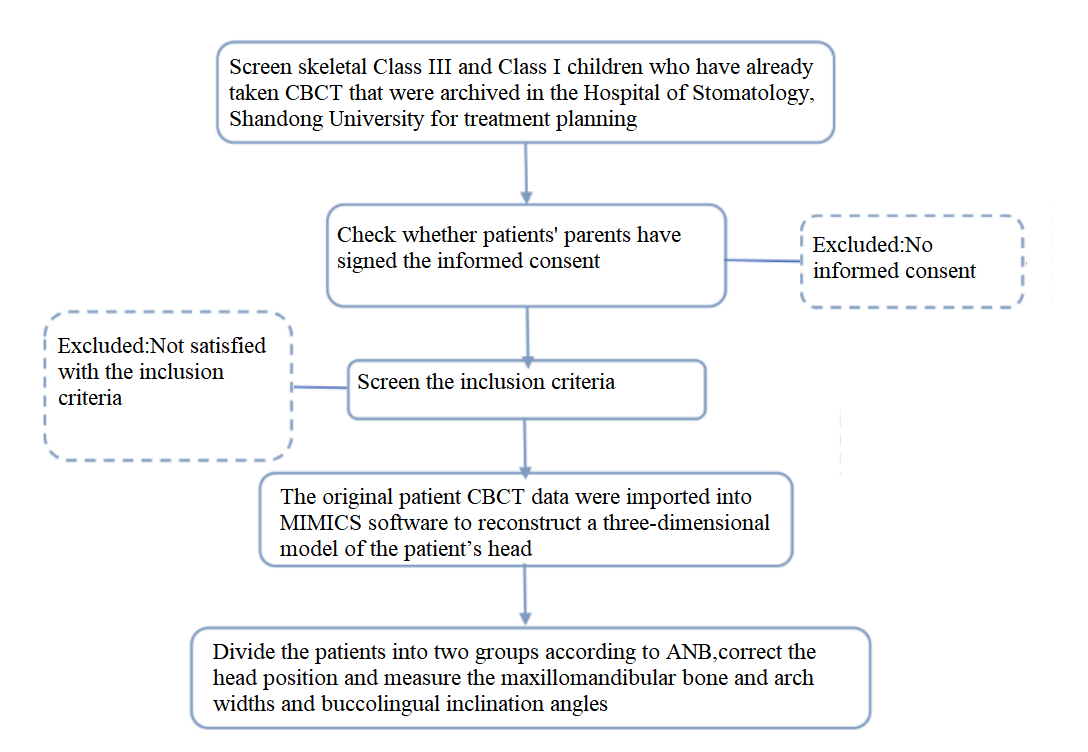 |
| Descriptive data | 14* | (a) Give characteristics of study participants (eg demographic, clinical, social) and information on exposures and potential confounders | 18 | Table 1 Distribution of sex and age   \| Variable \| Class III \| Class I \| Total \| \| --- \| --- \| --- \| --- \| \| Sex**,** n (%) \|  \|  \|  \| \| Male \| 14 (46.7) \| 17 (56.7) \| 31 (51.7) \| \| Female \| 16 (53.3) \| 13 (43.3) \| 29 (48.3) \| \| Age (y) \|  \|  \|  \| \| Average (SD) \| 8.04 (0.79) \| 8.26 (0.81) \| 8.15 (0.82) \| |
|  |  | (b) Indicate number of participants with missing data for each variable of interest |  | 0 |
|  |  | (c) *Cohort study*—Summarise follow-up time (eg, average and total amount) |  |  |
| Outcome data | 15* | *Cohort study*—Report numbers of outcome events or summary measures over time |  |  |
|  |  | *Case-control study—*Report numbers in each exposure category, or summary measures of exposure |  | *30 cases for each group.* |
|  |  | *Cross-sectional study—*Report numbers of outcome events or summary measures |  |  |
| Main results | 16 | (*a*) Give unadjusted estimates and, if applicable, confounder-adjusted estimates and their precision (eg, 95% confidence interval). Make clear which confounders were adjusted for and why they were included |  | 95% confidence interval |
|  |  | (*b*) Report category boundaries when continuous variables were categorized | 5;6 | Case-control study—The samples were divided into the study group (with skeletal Class III malocclusion, N=30) and the control group (with skeletal Class I occlusion, N=30).  Patients in the study group who met the following criteria were included: (1) ANB angle [formed by the subspinale (A), nasion (N), and supramental (B)] less than 0°;  Patients in the control group were selected using the following criteria: (1) 1° < ANB < 4.5°[18]; |
|  |  | (*c*) If relevant, consider translating estimates of relative risk into absolute risk for a meaningful time period |  |  |

Continued on next page

| Other analyses | 17 | Report other analyses done—eg analyses of subgroups and interactions, and sensitivity analyses | 9 | The normality of the outcome data of widths and angles was examined using the Kolmogorov–Smirnov test with SPSS 25.0 (IBM SPSS Statistics 25.0) software. As all data conformed to a normal distribution, independent-sample t tests were performed to compare the two groups. A P value <0.05 indicated statistical significance. |
| --- | --- | --- | --- | --- |
| Discussion | | | | |
| Key results | 18 | Summarise key results with reference to study objectives | 11-12 | The results of our study show that in the skeletal Class III malocclusion without posterior crossbite group, the width of the basal bone in the maxillary first molar area was significantly narrower (P < 0.05) and the width of the basal arch bone in the mandibular first molar area was significantly wider (P < 0.05) than that in the Class I occlusion group. A significant difference was found in the widths of the maxilla and mandible between the two groups (P < 0.01), which is similar to results reported for a population with permanent dentition by Tan et al[22].  The results showed no significant difference in the width of the upper or lower arch between the two occlusal types.  Our results showed that compensation of the transverse angle of the maxillary and maxillary permanent molars before orthodontic treatment is present in the period of mixed dentition in patients with skeletal Class III malocclusion without posterior crossbite. |
| Limitations | 19 | Discuss limitations of the study, taking into account sources of potential bias or imprecision. Discuss both direction and magnitude of any potential bias | 13 | The limitation of this study was that it included children with bony class III malformation without posterior teeth. The classification of bony class III malocclusion is complex, including not only the type of underdevelopment of the maxilla but also normal development of the mandible and the type of overdevelopment of the mandible. In addition, large variations in growth and development exist among different individuals and types of dentition replacement among 7- and 9-year-old children. Therefore, the samples are selected according to the development of similar bones and teeth. The characteristics of basal bone and dental arch width in patients with skeletal class Ⅲ malformation should be explored for better results. |
| Interpretation | 20 | Give a cautious overall interpretation of results considering objectives, limitations, multiplicity of analyses, results from similar studies, and other relevant evidence | 11-12 | This indicates that patients with skeletal Class III malocclusion without posterior crossbite in the early stage of mixed dentition also suffer from insufficient maxillary width and/or excessive mandibular width; in other words, uncoordinated maxillary and mandibular widths emerge during this period.  The patients in this study showed no crossbite of the posterior teeth but did show underdevelopment of the maxilla and/or overdevelopment of the mandible, indicating that during a clinical diagnosis, measurement of the dental arch width alone cannot fully reflect the coordination of the transverse widths of the upper and lower jaws; indeed, measurement of the width of the maxillary and mandibular bases might be more clinically significant.  After measuring the buccal and lingual inclination in the molar areas of children in the early stage of mixed dentition, we showed that the first permanent maxillary molars of children with skeletal Class III malocclusion without posterior crossbite compensated more for the buccal inclination than those of children with Class I occlusion. |
| Generalisability | 21 | Discuss the generalisability (external validity) of the study results | 13 | In clinical practice, rapid maxillary expansion (RME) is the gold standard treatment for the correction of maxillary transverse deficiency in the early mixed dentition stage[30]. RME applies force on teeth and alveolar processes through activation of the expansion screw and, as a result, promotes opening of the midpalatal suture and widening of the maxilla and its associated structures[31]. Interestingly, however, McNamara[29] found that following RME, there is expansion not only of the maxillary dental arch but also of the mandibular dental arch. Using CBCT, our study revealed transverse maxillomandibular discrepancy in patients with early mixed dentition and skeletal Class III malocclusion without posterior crossbite. The results suggest that for patients with early mixed dentition and skeletal Class III malocclusion, an efficient and effective treatment method is expanding the arch by RME to improve the width of the maxillary basal bone and synchronize the development of the upper and lower jaws, even in the absence of crossbite. |
| Other information | |  | | |
| Funding | 22 | Give the source of funding and the role of the funders for the present study and, if applicable, for the original study on which the present article is based | 14-15 | This study was funded by the National Natural Science Foundation of China (No. 81571010), the Key Clinical Research of Shandong University (No. 2020SDUCRCA 005), the Shandong University Graduate Education and Teaching excellent achievement training program (No. ZY2019004), the National Natural Science Foundation of China (No. 81701008), the Key Research and Development Program of Shandong Province, China (No. 2019GSF108187), and the Undergraduate Teaching Reform and Research Project of Shandong University Cheeloo Medical College (No. qlyxjy-202027). |

*Give information separately for cases and controls in case-control studies and, if applicable, for exposed and unexposed groups in cohort and cross-sectional studies.

**Note:** An Explanation and Elaboration article discusses each checklist item and gives methodological background and published examples of transparent reporting. The STROBE checklist is best used in conjunction with this article (freely available on the Web sites of PLoS Medicine at http://www.plosmedicine.org/, Annals of Internal Medicine at http://www.annals.org/, and Epidemiology at http://www.epidem.com/). Information on the STROBE Initiative is available at [www.strobe-statement.org](http://www.strobe-statement.org).
